# Trifluridine/Tipiracil Plus Bevacizumab for Vulnerable Patients With Pretreated Metastatic Colorectal Cancer: A Retrospective Study (WJOG14520G)

**DOI:** 10.1093/oncolo/oyad296

**Published:** 2023-11-10

**Authors:** Yosuke Kito, Hisato Kawakami, Seiichiro Mitani, Shinichi Nishina, Toshihiko Matsumoto, Takao Tsuzuki, Yudai Shinohara, Hozumi Shimokawa, Ryosuke Kumanishi, Takashi Ohta, Hiroo Katsuya, Takeshi Kawakami, Tomohiro Nishina, Hiroko Hasegawa, Kohei Akiyoshi, Yasutaka Chiba, Kentaro Yamazaki, Shuichi Hironaka, Kei Muro

**Affiliations:** Department of Medical Oncology, Ishikawa Prefectural Central Hospital, Ishikawa, Japan; Department of Medical Oncology, Kindai University Faculty of Medicine, Osaka, Japan; Department of Medical Oncology, Kindai University Faculty of Medicine, Osaka, Japan; Department of Medical Oncology, Kurashiki Central Hospital, Okayama, Japan; Department of Medical Oncology, Kobe City Medical Center General Hospital, Hyogo, Japan; Department of Internal Medicine, Japanese Red Cross Society Himeji Hospital, Hyogo, Japan; Department of Hematology/Oncology, Japan Community Healthcare Organization Kyushu Hospital, Fukuoka, Japan; Department of Hematology/Oncology, Japan Community Healthcare Organization Kyushu Hospital, Fukuoka, Japan; Department of Clinical Oncology, Aichi Cancer Center Hospital, Aichi, Japan; Department of Clinical Oncology, Kansai Rosai Hospital, Hyogo, Japan; Division of Hematology, Respiratory Medicine, and Oncology, Department of Internal Medicine, Faculty of Medicine, Saga University, Saga, Japan; Division of Gastrointestinal Oncology, Shizuoka Cancer Center, Shizuoka, Japan; Department of Gastrointestinal Medical Oncology, National Hospital Organization Shikoku Cancer Center, Ehime, Japan; Department of Gastroenterology and Hepatology, National Hospital Organization Osaka National Hospital, Osaka, Japan; Department of Medical Oncology, Osaka City General Hospital, Osaka, Japan; Clinical Research Center, Kindai University Hospital, Osaka, Japan; Division of Gastrointestinal Oncology, Shizuoka Cancer Center, Shizuoka, Japan; Department of Medical Oncology, Kyorin University Faculty of Medicine, Tokyo, Japan; Department of Clinical Oncology, Aichi Cancer Center Hospital, Aichi, Japan

**Keywords:** metastatic colorectal cancer, vulnerable patients, intensive therapy, trifluridine/tipiracil, bevacizumab

## Abstract

**Background:**

Trifluridine/tipiracil (FTD/TPI) plus bevacizumab has shown clinical benefit for metastatic colorectal cancer (mCRC) refractory to standard therapy. However, few data have been available for patients with pretreated mCRC who are intolerant of intensive therapy (vulnerable).

**Methods:**

We performed a multicenter retrospective study (WJOG14520G; TWILIGHT) of FTD/TPI plus bevacizumab for vulnerable patients with pretreated mCRC. Eligibility criteria included previous chemotherapy (although patients treated with all key cytotoxic agents, a fluoropyrimidine, oxaliplatin, and irinotecan, were excluded) and intolerance of full-dose combination therapy with oxaliplatin or irinotecan at the start of FTD/TPI plus bevacizumab.

**Results:**

The median age of 93 evaluable patients was 79 years (range, 21-90). Intolerance of intensive therapy was attributable to an older age in 60 (65%) patients, serious concomitant disease in 24 (26%) patients, and a poor performance status in 19 (20%) patients. FTD/TPI plus bevacizumab was administered as second-line treatment in 74 (80%) patients and as third- or fourth-line treatment in 19 (20%) patients. The objective response rate was 4.9% (95% confidence interval [CI], 1.4%-12.2%), and the disease control rate was 67.9% (95% CI, 56.6%-77.8%). With a median follow-up time of 21.6 months, median overall survival and progression-free survival were 18.6 months (95% CI, 12.1-23.2) and 6.3 months (95% CI, 5.0-8.3), respectively. Neutropenia of grade ≥3 developed in 50 (54%) patients, whereas 2 (2%) patients experienced febrile neutropenia, and no treatment-related death was observed.

**Conclusion:**

Our data show the potential efficacy and acceptable safety profile of FTD/TPI plus bevacizumab for vulnerable patients with pretreated mCRC.

## Teaser

This study evaluated efficacy and safety of trifluridine/tipiracil plus bevacizumab for patients with previously treated metastatic colorectal cancer who were intolerant of intensive therapy (vulnerable).

**Implications for Practice**

This was a multicenter retrospective study to evaluate efficacy and safety of trifluridine/tipiracil (FTD/TPI) plus bevacizumab for patients with previously treated metastatic colorectal cancer (mCRC) who were intolerant of intensive therapy (vulnerable). Based on phase III studies, FTD/TPI plus bevacizumab has been a standard therapy for patients previously treated with a fluoropyrimidine, oxaliplatin, and irinotecan, and a feasible alternative to capecitabine plus bevacizumab for previously untreated patients who are ineligible for intensive therapy. Results indicate this regimen is a possible treatment option for vulnerable patients with pretreated mCRC.

## Introduction

Colorectal cancer is the third most diagnosed cancer type and the second leading cause of cancer-related death worldwide.^1^ The standard of care for first- and second-line treatment of unresectable metastatic colorectal cancer (mCRC) has been a doublet (or triplet) chemotherapy regimen consisting of a fluoropyrimidine and either oxaliplatin or irinotecan (or all these cytotoxic agents) plus a molecularly targeted agent. However, a substantial proportion of patients is unsuitable for full-dose standard chemotherapy as a result of various factors including an older age, the presence of comorbidities, a poor performance status, a low tumor burden, and patient preference. Several clinical trials have been conducted for first-line treatment of patients who are not candidates for intensive therapy. In the case of elderly patients with previously untreated mCRC, a survival benefit has been demonstrated for the addition of bevacizumab to capecitabine,^2^ whereas the addition of oxaliplatin or irinotecan to a fluoropyrimidine was not associated with survival prolongation.^3-6^ For individuals with tumors wild-type for *RAS* who are frail or elderly (or both), single-arm phase II studies have detected a clinical benefit of monotherapy with an antibody to epidermal growth factor receptor (EGFR).^7-9^ On the basis of these findings, Japanese guidelines as well as the guidelines of the European Society for Medical Oncology and the National Comprehensive Cancer Network recommend a fluoropyrimidine plus bevacizumab, or anti-EGFR monotherapy in the case of tumors wild-type for *RAS*, as first-line treatment for patients who are unable to tolerate full-dose combination therapy with a fluoropyrimidine plus oxaliplatin or irinotecan (so-called “vulnerable patients”).^10-12^ However, little is known about second or later lines of treatment for vulnerable patients, some of whom may have become intolerant of intensive therapy after first-line treatment.

Trifluridine/tipiracil (FTD/TPI) is an orally administered combination of a thymidine-based nucleic acid analog (trifluridine) and a thymidine phosphorylase inhibitor (tipiracil hydrochloride). A phase III study (RECOURSE) revealed a significant survival benefit of FTD/TPI compared with placebo for patients with refractory mCRC.^13^ The efficacy and safety of adding bevacizumab to FTD/TPI were subsequently demonstrated in several phase II studies in this setting.^14-17^ The SUNLIGHT phase III trial recently showed the superiority of FTD/TPI plus bevacizumab over FTD/TPI alone in patients previously treated with a fluoropyrimidine, oxaliplatin, and irinotecan, offering a new standard therapy for this population.^18^ Given its relatively low frequency of nonhematologic toxicities, the efficacy and safety of the combination of FTD/TPI plus bevacizumab have also been evaluated in patients unsuitable for intensive therapy. The SOLSTICE phase III trial was thus designed to examine the superiority of FTD/TPI plus bevacizumab over capecitabine plus bevacizumab as first-line treatment for these patients.^19^ Although FTD/TPI plus bevacizumab failed to achieve superiority, this regimen showed similar efficacy to capecitabine plus bevacizumab with no new safety concerns, suggesting that FTD/TPI plus bevacizumab might be a feasible option for subsequent treatment of this population. Given the lack of data addressing this clinical question, we have now retrospectively investigated the efficacy and safety of FTD/TPI plus bevacizumab for vulnerable patients with previously treated mCRC in the clinical practice setting.

## Patients and Methods

### Study Design and Patient Selection

West Japan Oncology Group (WJOG) 14520G (TWILIGHT) is a multicenter, retrospective, observational study of FTD/TPI plus bevacizumab as second- or later-line treatment for vulnerable patients with mCRC who are intolerant of intensive chemotherapy. The main inclusion criteria were as follows: (1) histologically confirmed colorectal adenocarcinoma, (2) age of ≥20 years, (3) Eastern Cooperative Oncology Group (ECOG) performance status of 0 to 2, (4) previous treatment with chemotherapy for unresectable disease (but excluding patients with a history of having received all key cytotoxic agents: a fluoropyrimidine, oxaliplatin, and irinotecan), (5) treatment with FTD/TPI plus bevacizumab between May 2014 and October 2020, and (6) intolerance of intensive therapy (defined as full-dose combination chemotherapy with a fluoropyrimidine and either oxaliplatin or irinotecan) as a result of medical condition. Patients deemed unsuitable for intensive therapy simply because of a low tumor volume or personal preference were excluded.

The study protocol was approved by the WJOG Protocol Review Committee and the institutional review board of each participating institution, and it complied with the provisions of the Declaration of Helsinki as well as Ethical Guidelines for Medical and Health Research Involving Human Subjects in Japan.^20^ Consent to participation was obtained via an opt-out form on the website of WJOG and each participating institution. This study was registered in the University Hospital Medical Information Network (UMIN) Clinical Trials Registry (UMIN000044136).

### Treatment and Assessments

All patients were treated with the combination of FTD/TPI and bevacizumab. The approved dosage and administration of FTD/TPI in Japan were 35 mg/m^2^ orally twice daily on days 1 to 5 and 8 to 12 every 4 weeks. Bevacizumab was administered intravenously at a dose of 5 mg/kg every 2 weeks. Dose reductions and schedule modifications were instituted at the discretion of the physician.

Efficacy was assessed on the basis of overall survival (OS), progression-free survival (PFS), time to treatment failure (TTF), objective response rate (ORR), and disease control rate (DCR). OS was calculated from the initiation of treatment to death from any cause, PFS from the initiation of treatment to disease progression or death from any cause, and TTF from the initiation of treatment to the last administration of FTD/TPI or disease progression. OS, PFS, and TTF were evaluated by the Kaplan-Meier method. Tumor response was assessed according to Response Evaluation Criteria in Solid Tumors (version 1.1). ORR was defined as the proportion of patients whose best response was a complete response or a partial response, and DCR as the proportion of patients whose best response was a complete response, a partial response, or stable disease.

## Results

### Patient Characteristics

A total of 96 patients from 26 institutions was registered between July 2021 and January 2022. Given that 3 patients who did not meet the inclusion criteria were excluded, 93 patients were evaluated for the analysis. The baseline characteristics of these 93 patients are summarized in Table 1. The median age was 79 years (range, 21-90 years), 10 (11%) patients had an ECOG performance status of 2, and 32 (34%) patients had tumors known to be wild-type for *RAS*. Intolerance of intensive therapy was attributable to an older age in 60 (65%) patients, serious concomitant disease in 24 (26%) patients, and poor performance status in 19 (20%) patients (Table 2).

**Table 1.** Characteristics of the study patients.

Characteristic	*n* = 93	
Age (years), median (range)	79 (21-90)	
<75 years	31	33%
≥75 years	62	67%
Sex		
Male	49	53%
Female	44	47%
ECOG performance status		
0	23	25%
1	60	65%
2	10	11%
Location of primary tumor		
Right-sided	28	30%
Left-sided	65	70%
Primary tumor resection		
Yes	80	86%
No	13	14%
Pre- or postoperative chemotherapy		
Yes	35	38%
No	58	62%
Prior chemotherapy agent for metastatic disease		
Fluoropyrimidine	93	100%
Oxaliplatin	46	49%
Irinotecan	14	15%
Antiangiogenic antibodies	84	90%
Anti-EGFR antibodies	18	19%
Treatment line		
Second	74	80%
Third or fourth	19	20%
*RAS* status		
Wild type	32	34%
Mutant	59	63%
Unknown	2	2%
*BRAF* status		
Wild type	46	49%
Mutant	1	1%
Unknown	46	49%
MSI/MMR status		
MSI-high or MMR-deficient	3	3%
MSS or MMR-proficient	56	60%
Unknown	34	37%

Values are *n* (%) with the exception of median (range) age.

Abbreviaitons: ECOG: Eastern Cooperative Oncology Group; EGFR: epidermal growth factor receptor; MSI, microsatellite instability; MMR, mismatch repair; MSS, microsatellite stable.

**Table 2.** Reasons for intolerance of intensive therapy in the study patients.

Reason for intolerance of intensive therapy	*n* = 93	
Older age	60	65%
Serious concomitant disease^*^	24	26%
Poor performance status	19	20%
Renal dysfunction	5	5%
Adverse event in prior therapy	4	4%
Liver dysfunction	3	3%
Hypocytosis	3	3%
Other	7	8%
Number of reasons		
1	62	67%
≥2	31	33%

^*^Including pulmonary disease (*n* = 16), malignant disease (*n* = 4), cardiac disease (*n* = 3), metabolic disease (*n* = 1), mental disease (*n* = 1), paralysis due to poliomyelitis (*n* = 1), and postchemotherapy for lymphoma (*n* = 1).

With regard to prior treatment for metastatic disease, 93 (100%) patients received a fluoropyrimidine, whereas 46 (49%) and 14 (15%) patients received oxaliplatin and irinotecan, respectively. Although *BRAF* mutation and microsatellite instability, high/mismatch repair, deficient status were detected in 1 and 3 patients, respectively, no patient had previously received a BRAF inhibitor or an immune checkpoint inhibitor. FTD/TPI plus bevacizumab was administered as second-line treatment in 74 (80%) patients and as third- or fourth-line treatment in 19 (20%) patients. The proportions of patients with *RAS* mutation-positive tumors (70% vs. 37%), who received oxaliplatin for pre- or postoperative treatment (18% vs. 0%), or who received oxaliplatin or irinotecan in first-line treatment (61% vs. 32%) were numerically higher for the second-line setting than for the third- or fourth-line setting. Thirty-three patients had no history of either oxaliplatin or irinotecan treatment for metastatic disease, 31 (94%) of whom were aged 75 years or older.

### Efficacy

**Figure 1.**
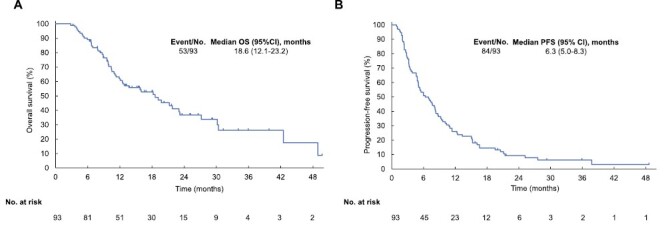
Kaplan-Meier survival analysis. (**A**) overall survival (OS). (**B**) Progression-free survival (PFS). Abbreviation: CI, confidential interval.

The median OS was 18.6 months (95% confidence interval [CI], 12.1-23.2 months), with 53 events of death, and the median PFS was 6.3 months (95% CI, 5.0-8.3 months), with 84 events of disease progression, at a median follow up of 21.6 months (Fig. 1). The median TTF was 4.5 months (95% CI, 3.3-7.1 months). Among 81 patients with measurable lesions, ORR and DCR were 4.9% (95% CI, 1.4-12.2%) and 67.9% (95% CI, 56.6-77.8%), respectively.

**Figure 2.**
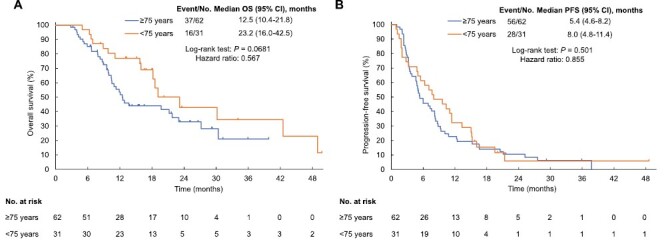
Kaplan-Meier survival analysis. (**A**) overall survival (OS) according to age. (**B**) Progression-free survival (PFS) according to age. Abbreviation: CI, confidential interval.

**Figure 3.**
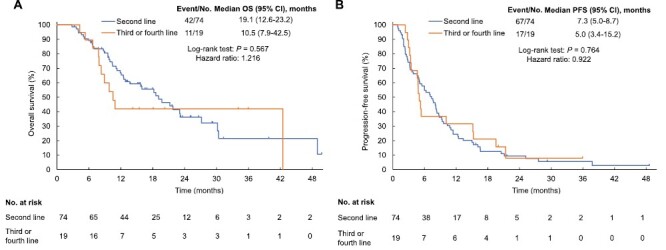
Kaplan-Meier survival analysis. (**A**) overall survival (OS) according to treatment line. (**B**) Progression-free survival (PFS) according to treatment line. Abbreviation: CI, confidential interval.

Subgroup analysis according to age revealed that OS (median of 12.5 vs. 23.2 months; hazard ratio [HR] of 0.57; *P* = .068) and PFS (median of 4.1 vs. 7.1 months; HR of 0.65; *P* = .064) tended to be shorter for patients aged ≥75 years than for those aged <75 years (Fig. 2). Subgroup analysis according to treatment line (second line vs. third or fourth line) revealed no significant difference in OS (median of 19.1 vs. 10.5 months, respectively; HR of 1.22; *P* = .57) or PFS (median of 7.3 vs. 5.0 months, respectively; HR of 0.92; *P* = .76) (Fig. 3). ORR was similar in subgroup analyses by age (3.6% vs. 7.7%, respectively; *P* = .59) and treatment line (4.7% vs. 5.9%, respectively; *P* = 1.00).

### Safety

Neutropenia of grade ≥3 occurred in 50 (54%) patients, whereas there were 2 (2%) patients with febrile neutropenia and no treatment-related deaths. The other adverse events of grade ≥3 included anemia (20%), thrombocytopenia (9%), hypertension (6%), proteinuria (4%), anorexia (3%), and diarrhea (3%) (Table 3). A total of 30 (32%) patients received a reduced dose of FTD/TPI at the initial cycle. There were no significant differences in adverse events between patients with or without a reduced dose of FTD/TPI at the initial cycle. Eighty-nine patients discontinued treatment as a result of disease progression (72 [77%] patients), patient refusal (10 [11%] patients), adverse events (6 [6%] patients), or lost contact (1 [1%] patients).

**Table 3.** Adverse events in the study patients (*n* = 93).

Event	Any grade		Grade ≥3	
Neutropenia	75	81%	50	54%
Anemia	75	81%	19	20%
Thrombocytopenia	53	57%	8	9%
Fatigue	63	68%	1	1%
Anorexia	59	63%	3	3%
Nausea	39	42%	1	1%
Diarrhea	30	32%	3	3%
Mucositis oral	21	23%	0	0%
Febrile neutropenia	2	2%	2	2%
Hypertension	45	48%	6	6%
Proteinuria	57	61%	4	4%
Bowel perforation	1	1%	1	1%
Bleeding	5	5%	1	1%
Thromboembolic event	1	1%	0	0%

Values are *n* (%).

### Subsequent Chemotherapy

Of the 89 patients who discontinued FTD/TPI plus bevacizumab, 42 individuals received subsequent chemotherapy. The regimens in total of subsequent therapies included regorafenib (16 patients), doublet regimens (13 patients), anti-EGFR monotherapy (7 patients), and irinotecan with or without biologics (6 patients). The doublet regimens were FOLFIRI (5 patients), FOLFOX (4 patients), IRIS (S-1 and irinotecan, 3 patients), FOLFOX (3 patients), and CAPOX (capecitabine and oxaliplatin, 2 patients) with or without biologics. No patient received an immune checkpoint inhibitor or a BRAF inhibitor as subsequent therapy.

## Discussion

Our retrospective study has shown that treatment with the combination of FTD/TPI and bevacizumab was associated with a median OS and PFS of 18.6 and 6.3 months, respectively, and with moderate toxicity for vulnerable patients with previously treated mCRC. These data are similar to those for the recent TRUSTY phase III trial, which investigated the noninferiority of FTD/TPI plus bevacizumab in comparison with full-dose combination therapy with a fluoropyrimidine and irinotecan plus bevacizumab as second-line treatment for mCRC.^21^ In the present study, although 80% of patients were treated in the second-line setting, there was no significant difference in efficacy between the second-line and the third- or fourth-line settings.

Evidence do not support the use of either oxaliplatin or irinotecan in combination with fluoropyrimidine plus bevacizumab in elderly or vulnerable patients with mCRC in the first-line setting.^3-6^ However, few data regarding second- or later-line treatment have been available to date. We recently reported real-world data for treatment sequencing in vulnerable patients with mCRC in a multicenter retrospective survey.^22^ In this survey, among 195 vulnerable patients who progressed on first-line therapy consisting of “less-intensive regimens” (defined as a fluoropyrimidine with or without biologics, reduced-dose doublet regimens with or without biologics, or anti-EGFR monotherapy), 74 (38%) individuals received less-intensive regimens as second-line treatment, achieving a median OS and PFS of 13.7 and 5.5 months, respectively. Interestingly, there was no survival difference between patients received a fluoropyrimidine with or without biologics versus those received reduced-dose doublet regimens with or without biologics in the second-line setting as well as the first-line setting, suggesting that the addition of oxaliplatin or irinotecan is not always necessary for elderly or vulnerable patients with mCRC in second-line treatment and beyond. Furthermore, for patients who are never able to use oxaliplatin or irinotecan, the second-line treatment after the first-line fluoropyrimidine with or without biologics must be the best supportive care, of which the prognosis was shown to be dismal (3.5 months).^22^ In this regard, our data thus offer an important treatment option for those population, given that the efficacy of FTD/TPI plus bevacizumab in the present study appears to be better than that of second-line less-intensive regimens in our previous study, although the patient backgrounds differed between the 2 studies.

The present study excluded patients who were ineligible for intensive therapy because of a low tumor burden or patient preference, with such individuals tending to have a good prognosis, although the SOLSTICE trial included these patients.^19^ In the Japanese treatment guidelines,^11^ “vulnerable patients” are defined as those who are considered intolerant of first-line combination therapy with oxaliplatin or irinotecan. The inclusion criteria for the present study were established on the basis of this definition. Some vulnerable patients might receive FTD/TPI plus bevacizumab after first-line treatment with a less intensive regimen, whereas others might become vulnerable after progression on first-line intensive therapy with oxaliplatin or irinotecan. To evaluate the efficacy and safety of FTD/TPI plus bevacizumab for vulnerable patients with pretreated mCRC, we adopted criteria that excluded patients with a history of having received all key cytotoxic agents (a fluoropyrimidine, oxaliplatin, and irinotecan) but which included those intolerant of intensive therapy as a result of medical condition. Despite the worse background of patients in our study compared with those in the first-line SOLSTICE trial and the second-line TRUSTY trial, the present study showed a good efficacy and acceptable safety profile for FTD/TPI plus bevacizumab. These findings suggest that FTD/TPI plus bevacizumab can be a preferred regimen in second or later lines of treatment for vulnerable patients with mCRC.

Our study has several limitations. First, it was retrospective in design and included a relatively small number of patients. Given that the vulnerable patients were highly heterogeneous, it might also have been affected by selection bias. Furthermore, the patients were treated with various drug doses and treatment schedules for the FTD/TPI and bevacizumab combination therapy. Second, the impact of subsequent chemotherapies including doublet regimens on the OS could not be evaluated because of the luck of sufficient data. Third, it was difficult to evaluate the efficacy of FTD/TPI plus bevacizumab for the vulnerable patients with previously treated mCRC in our study because of the lack of appropriate reference data, such as outcome of chemotherapy or best supportive care in such patients. We conducted our multicenter retrospective survey and previously reported the reference data to address this deficiency.^22^ Prospective comparative studies will be needed to fully resolve this issue. Finally, all patients enrolled in the present study were Japanese. However, no ethnic differences in the efficacy or safety of FTD/TPI plus bevacizumab between Japanese and Western patients have been described previously.^13,23^

## Conclusion

Our retrospective study has revealed a promising clinical activity with acceptable toxicity of FTD/TPI plus bevacizumab mostly as second-line treatment for vulnerable patients with previously treated mCRC who are intolerant of intensive therapy, and it, therefore, indicates that this regimen is a possible treatment option for such patients.

## Data Availability

The data underlying this article will be shared on reasonable request to the corresponding author.
